# Exploration of the feasibility to combine patients with chronic obstructive pulmonary disease and chronic heart failure in self-management groups with focus on exercise self-efficacy

**DOI:** 10.1080/02813432.2022.2073961

**Published:** 2022-05-16

**Authors:** Maaike Giezeman, Kersti Theander, Ann-Britt Zakrisson, Josefin Sundh, Mikael Hasselgren

**Affiliations:** a Faculty of Medicine and Health, Örebro University, Örebro, Sweden; b Centre for Clinical Research, Region Värmland, Karlstad, Sweden; c Department of Respiratory Medicine, School of Medical Sciences, Örebro University, Örebro, Sweden

**Keywords:** Pulmonary disease, chronic obstructive, heart failure, self-efficacy, exercise, self-management, feasibility studies

## Abstract

**Objective:**

To compare the level of exercise self-efficacy, symptoms, functional capacity and health status and investigate the association between these variables in patients with chronic obstructive pulmonary disease (COPD) and chronic heart failure (CHF). Additionally, to investigate how diagnosis, symptoms and patient characteristics are associated with exercise self-efficacy in these patient groups.

**Design:**

Cross-sectional study.

**Setting:**

Primary care.

**Subjects:**

Patients (*n* = 150) with COPD (*n* = 60), CHF (*n* = 60) and a double diagnosis (*n* = 30).

**Main outcome measures:**

Swedish SCI Exercise Self-Efficacy score, modified Medical Research Council Dyspnea score (mMRC), fatigue score, pain severity score, Hospital Anxiety and Depression Scale, functional capacity measured as six-minute walking distance and health status measured by a Visual Analogue Scale.

**Results:**

Levels of exercise self-efficacy, health status and symptoms were alike for patients with COPD and patients with CHF. Functional capacity was similar after correction for age. Associations with exercise self-efficacy were found for slight dyspnea (mMRC = 1) (R −4.45; 95% CI −8.41– −0.50), moderate dyspnea (mMRC = 2) (−6.60;−10.68– −2.52), severe dyspnea (mMRC ≥ 3) (−9.94; −15.07– −4.80), fatigue (−0.87;−1.41– −0.32), moderate pain (−3.87;−7.52– −0.21) and severe pain (−5.32;−10.13– −0.52), symptoms of depression (−0.98;−1.42– −0.55) and anxiety (−0.65;−0,10– −0.32), after adjustment for diagnosis, sex and age.

**Conclusion and implications:**

Patients with COPD or CHF have similar levels of exercise self-efficacy, symptoms, functional capacity and health status. More severe symptoms are associated with lower levels of exercise self-efficacy regardless of diagnosis, sex and age. When forming self-management groups with a focus on exercise self-efficacy, it seems more relevant to consider level of symptoms than the specific diagnosis of COPD or CHF.

**Key points**
- Exercise training is an important part of self-management in patients with COPD and chronic heart failure (CHF). High exercise self-efficacy is required for optimal exercise training.
- Patients with COPD and CHF have similar symptoms and similar levels of exercise self-efficacy, functional capacity and health status.
- Not the diagnosis, but symptoms of dyspnea, fatigue, pain, depression and anxiety are important factors influencing exercise self-efficacy and need to be addressed.
- When forming self-management groups with a focus on exercise self-efficacy, it seems more relevant to consider the level of symptoms than the specific diagnosis of COPD or CHF.

## Introduction

Chronic heart failure (CHF) and chronic obstructive pulmonary disease (COPD) are common chronic diseases in elderly people and mainly managed in primary health care [1–3]. Patients with COPD and CHF have many symptoms and functional limitations in common. Besides the cardinal symptoms of dyspnea and fatigue, even symptoms of anxiety, depression and pain are common in patients with these diseases [4]. Management of COPD and CHF aims to prevent disease progress and periods of deterioration or exacerbation and to maintain the best possible health. Besides pharmaceutical treatment, support is needed to optimize patients’ self-management. An important part of self-management for both conditions is to perform exercise training [5,6]. Training has been shown to reduce and prevent muscle deconditioning, improve the patient’s health and decrease the need for hospitalization [5,7].

Pulmonary and cardiac rehabilitation programs, aimed at helping the patient to improve self-management, are well-established in hospital settings but less common in primary health care [8,9]. Lately, a more symptom- and disability-focused rehabilitation has been advocated instead of a strictly diagnosis-oriented approach [10]. It has also been stated that exercise training is a core component in the rehabilitation program, but that even other interventions to address symptoms, education and psychological needs should be included [10]. An important psychological factor that can be met is the patient’s self-efficacy.

Self-efficacy is defined as the confidence that one has in the ability to perform a specific action in a specific situation [11]. Reaching a behavioral goal is considered to be less about skills and more about the person’s beliefs about what can be done with these skills in challenging situations. Self-efficacy beliefs can be general, tied to a certain disease or more specific to an action. Exercise self-efficacy is the specific self-efficacy referring to the confidence a patient has to be physically active. A high exercise self-efficacy is a prerequisite for optimal exercise behavior. This has been found to be true in both patients with COPD and CHF [12,13].

According to the self-efficacy theory, four factors influence an individual's level of self-efficacy: past successful or unsuccessful performance, verbal persuasion by a trusted person, emotions caused by physiological response to the exercise and ‘modeling’ [11,14]. Modeling means that a patient’s self-efficacy can increase by seeing or hearing someone in the same situation solving problems and succeeding. It is a powerful tool in self-management education and its positive effect can be achieved in group-oriented programs [14].

**Figure 1.**
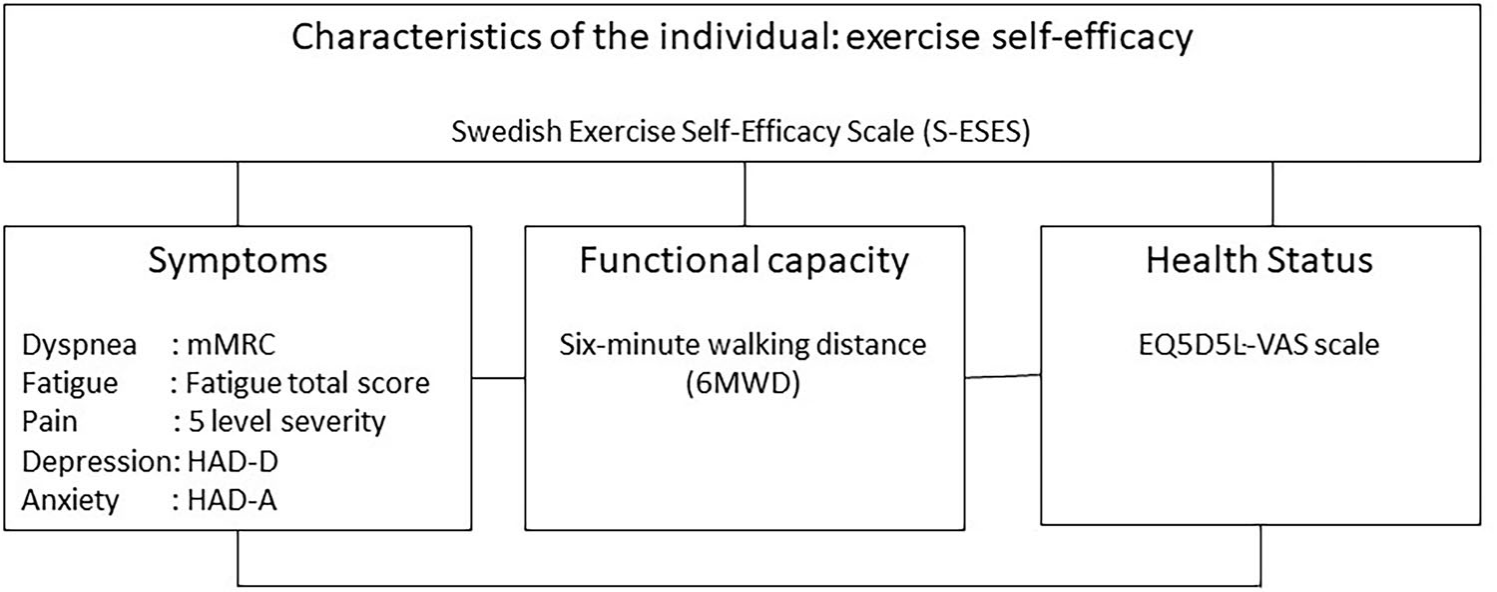
Conceptual model for the relation between variables in patients with chronic heart failure (CHF) and chronic obstructive pulmonary disease (COPD), with indication of the used measuring instruments. mMRC: modified Medical Research Council Dyspnea scale; HAD: Hospital Anxiety and Depression Scale, EQ5D5L-VAS: Visual Analogue Scale of the EuroQol group.

Patients with COPD and CHF share symptomatology, the diseases often coexist, and the exercise recommendations are similar [5,6,15]. Therefore, it seems logical to assume a beneficial modeling effect when patients with CHF and COPD are enrolled in the same self-management education group with a focus to increase exercise self-efficacy. Little is known, though, about whether the level of exercise self-efficacy in these groups is similar. Self-efficacy has been studied in both patients with COPD and CHF, but levels are hard to compare because these studies often focused on general self-efficacy or used disease-specific instruments. It is also important to know whether the relation between exercise self-efficacy and other health parameters, like symptoms, functional capacity and health status, is comparable for patients with COPD and CHF. To investigate the relation between these health parameters in a primary health care population, we used a conceptual model based on the revised Wilson and Cleary model [16]. Our conceptual model describes the relation of five frequently present symptoms in both COPD and CHF with physical capacity and health status. This pathway is, on every component, related to exercise self-efficacy as an important characteristic of the patient (Figure 1).

The aim of this study is to explore the feasibility of joint self-management groups with a focus on exercise self-efficacy for patients with COPD and CHF by comparing the level of exercise self-efficacy, symptoms, functional capacity and health status and investigating the correlation between these variables in primary health care. Additionally, to investigate how symptoms and patient characteristics are associated with exercise self-efficacy in these patient groups.

## Material and methods

### Study population

This study used the baseline data collected from 150 patients enrolled in the Symptoms and Function Study (SAFS), an intervention study testing a joint self-management group-intervention for patients with COPD and CHF recruited in primary health care, with the goal to increase the patient’s self-efficacy. The SAFS study has been described in detail earlier [17]. Patients with a spirometry-verified COPD, or ICD-10 code for heart failure (I50), were consecutively selected from nine primary health care centers in Sweden. To be included, the patients should have at least one symptom of dyspnea, fatigue, sleeping disorder, or pain. Moreover, the patients should be in a stable state; if they had an exacerbation of their COPD or a heart infarction in the last three months, they were excluded. Additional exclusion criteria were patients living in nursing homes, with mental impairment and insufficient knowledge of the Swedish language. Having both diagnoses was not an exclusion criterion for participating in the group-education sessions. Patients who, in the questionnaire, answered that they had both diseases, or declared having the diagnosis other than what they were selected for, were considered to have a patient-reported double diagnosis. In this way, we had a group of 150 patients, whereof 60 had a doctor’s diagnosis of COPD, 60 had a doctor’s diagnosis of CHF and 30 had a patient-reported double diagnosis.

### Measurements

#### Characteristics of the individual

Data from the written questionnaire contained information about age, sex, whether they lived alone, level of physical activity and smoking habits. For patients with CHF, the New York Heart Association Classification Scale (NYHA) was included. The NYHA classification has four points ranging from 1 = no symptoms to 4 = symptoms in rest.

Exercise self-efficacy was measured using the Swedish validated version of the SCI Exercise Self-Efficacy Scale (S-ESES) [18]. This scale consists of 10 statements about how confident the person is about performing exercise in different situations, for example, when feeling tired or without support of family and friends. The statements are scored on a four-point Likert scale where 1 = not at all true; 2 = rarely true, 3 = moderately true and 4 = always true. The total score ranges from 10 to 40, where 40 indicates the highest level of exercise self-efficacy. The Cronbach’s alpha for the S-ESES items in this study was 0.95.

#### Symptoms

Dyspnea was assessed by the modified Medical Research Council Dyspnea Scale (mMRC). The mMRC has five points ranging from 0 = I only get breathless with strenuous exercise, to 4 = too breathless to leave the house or breathless when dressing or undressing [19].

Fatigue was assessed with three questions and calculated into a total score for frequency, duration and severity (range 0–9) [20]. The frequency of fatigue for the past month was scored as 0= not a problem, 1 = 1–7 days a month, 2 = 8–14 days a month, 3 = 15–21 days a month and 4 = 22–30 days a month. The duration was scored as 0= no experience, 1= less than 6 h a day, 2 = 6–12 h a day and 3= more than 12 h a day. The severity of fatigue was scored as 0 = not a problem, 1= one of my less severe problems and 2= one of my worst symptoms.

Symptoms of depression and anxiety were assessed by the Swedish version of the Hospital Anxiety and Depression Scale (HAD). It consists of seven items in two subscales (score range 0–21) and measures the degree of anxiety (HAD-A) and depression (HAD-D). A higher score indicates higher level of depressive or anxiety symptoms. In clinical practice, a HAD score over 8 can be indicative of the presence of the respective mood disorder [21].

Severity of pain was assessed by using the patient’s answer to the question about their current level of pain or discomfort from the questionnaire that was developed by the EuroQol group (EQ5D5L). The question was answered on a five-point Likert scale ranging from 0 = I have no pain or discomfort to 4 = I have extreme pain or discomfort [22].

#### Functional capacity

The functional capacity of the patients was assessed by testing the six-minute walking distance (6MWD), when the patient had to walk as far as possible during 6 min, after receiving standardized instructions [23].

#### Health status

To assess health status, we used the Visual Analogue Scale from the EuroQol group (EQ5D5L-VAS). The patients were asked to score their perceived health today on a vertical VAS from 0 to 100, in which 0 is the worst and 100 the best imaginable health [22].

### Ethical consideration

This study was approved by the Research Ethics Committee in Uppsala (2012/189/1). Written informed consent was obtained from all participants.

### Statistical analysis

Categorical and nominal data are presented as frequencies and percentages, median and inter-quartile range; for continuous data, mean and standard deviations (SD) are used. Patients with a double diagnosis were excluded from the comparative analyses but included in the merged analysis. Differences between patients with COPD and CHF were analyzed with the chi-square test for proportions, Mann–Whitney U-test for non-parametric data or student’s t-test for parametric averages. Analyses were repeated with stratification for sex and age group. The age groups used were ≤73 and > 73 years; being the mean age in this study population. Patients with incomplete data on S-ESES (*n* = 21), fatigue total score (*n* = 16) HAD-D (*n* = 14) and HAD-A (*n* = 14) were excluded from the analyses including these variables. Spearman correlation coefficients were used to analyze the correlation between the different variables separately. This analysis was performed for the whole group (*n* = 150) and separately for the patients with COPD (*n* = 60) or CHF (*n* = 60). Bonferroni–Holm method was used to correct for the use of multiple variables [24]. For values of the correlation coefficient, 0–0.19 was regarded as very weak, 0.2–0.39 as weak, 0.40–0.59 as moderate, 0.6–0.79 as strong and 0.8–1 as very strong correlation [25].

To analyze what variables are associated with the level of exercise self-efficacy in the study population (*n* = 150), linear regression analysis was performed with the S-ESES as dependent variable and the different symptoms and patient characteristics as independent variables. These analyses were performed unadjusted and adjusted for diagnosis group (COPD, CHF, double diagnosis), sex and age. Diagnosis groups, mMRC, physical activity and pain were modeled as a series of binary dummy variables. The groups with mMRC = 3 and mMRC = 4 were merged for this analysis, since there were only three patients in the latter group. For our analyses, IBM SPSS statistics version 26 was used. A *p* value of <.05 was considered statistically significant.

## Results

### Patient characteristics

There were fewer men (*n* = 29) in the COPD group than in the CHF group (*n* = 46) and patients with COPD were, on average, younger. There were more daily smokers in the COPD group (Table 1). Exercise self-efficacy scores were similar for both groups (Table 2). No significant differences were found when stratified for sex and age groups (data not shown).

**Table 1.** Patient characteristics.

	All patients	COPD	CHF	COPD and CHF
	*n* = 150	*n* = 60	*n* = 60	*n* = 30
Men, *n* (%)	75 (50)	23 (38)	40 (67)*	12 (40)
Age, years, *mean (SD)*	73 (8)	69 (8)	78 (8)*	72 (6)
Living alone*, n* (%)	58 (39)	20 (34)	26 (43)	12 (40)
Employed, *n* (%)	20 (8)	14 (22)	5 (8)*	1 (3)
Current daily smoking, *n* (%)	27 (18)	17 (28)	5 (9)*	6 (21)
Physical activity, *n* (%)				
No	42 (28)	20 (35)	13 (22)	9 (32)
15 min/day	38 (26)	14 (24)	18 (31)	6 (21)
30 min/day	39 (27)	13 (22)	16 (27)	10 (36)
>30 min/day and at least once a week more intensive training	26 (18)	11 (19)	12 (20)	3 (11)
NYHA classification, *n* (%) *n =* 78				
I			3 (5)	2 (9)
II			25 (44)	9 (43)
IIIa			19 (33)	3 (14)
IIIb			8 (14)	6 (29)
IV			2 (4)	1 (5)

COPD: chronic obstructive pulmonary disease; CHF: chronic heart failure; NYHA: New York Heart Association; SD: standard deviation.

*Statistically significant difference between the COPD and CHF groups at a *p* < .05 level.

**Table 2.** Comparison of the level of exercise self-efficacy, symptoms, functional status and health status between patients with chronic obstructive pulmonary disease (COPD) and chronic heart failure (CHF).

	All patients	COPD	CHF
	*n* = 150	*n* = 60	*n* = 60
Swedish Exercise Self-Efficacy Scale, *mean (SD)*^a^ *n = 129*	23.0 (8.2)	24.5 (8.3)	22.5 (8.2)
mMRC, *n (%) n = 145*			
0	26 (17)	12 (20)	8 (14)
1	55 (37)	25 (42)	22 (40)
2	44 (29)	17 (29)	19 (34)
3	17 (11)	5 (9)	5 (9)
4	3 (2)	0 (0)	2 (3)
Fatigue total score^b^, *median (IQR) n = 134*	5 (2.75–6.25)	5 (2–6)	5 (3–7)
Depression score (HADS), *median (IQR)*^b^ *n = 136*	3 (1–5.75)	2 (1–5)	3 (1–6)
Anxiety score (HADS), *median* (IQR)^b^ *n = 136*	4 (1–7)	4 (2–8)	4 (1–8)
Severity of pain, *n (%) n = 146*			
No pain	30 (20)	13 (22)	10 (17)
Slight pain	41 (28)	17 (29)	18 (31)
Moderate pain	58 (40)	23 (40)	24 (42)
Severe pain	17 (12)	5 (9)	6 (10)
Extreme pain	0 (0)	0 (0)	0 (0)
Six-minute walking distance, meters, *mean (SD)*^b^ *n = 145*	379 (104)	409 (83)	356 (98)*
Health status, VAS score, *median (IQR)*^b^ *n = 144*	65 (50–80)	67.5 (50–78.5)	65 (50–80)

COPD: chronic obstructive pulmonary disease; CHF: chronic heart failure; SD: standard deviation; mMRC: modified Medical Research Council Dyspnea score; IQR: interquartile range; HADS: Hospital Anxiety and Depression Scale; VAS: Visual Analogue Scale.

^a^Student’s t-test.

^b^Mann–Whitney U-test.

*Statistically significant difference between the groups at a *p* < .05 level.

#### Symptoms

There was no significant difference in the level of symptoms between the two patient groups (Table 2). Of the patients with COPD and CHF, 38% and 46%, respectively, had an mMRC score ≥ 2. About one-third of all patients indicated that fatigue was one of their worst problems or their worst problem (data not shown) and the median fatigue total score was five on a scale of zero to nine. The HAD scores showed that the majority had no symptoms at the level that could indicate the presence of a diagnosis of depression or anxiety disorder. About half of the patients reported moderate to severe pain, but no patients reported extreme pain. No significant difference was found for men and women or age group for all symptom variables (data not shown).

#### Functional capacity

Patients with COPD had a higher mean 6MWD than patients with CHF, but after correction for age, this difference was no longer significant. There was no statistically significant difference in 6MWD for men and women (data not shown).

#### Health status

There was no statistically significant difference in the VAS score for perceived health between patients with CHF and COPD (Table 2) nor between men and women and age groups (data not shown).

#### Associations within our conceptual model

In patients with COPD, statistically significant weak to moderate correlation coefficients were found for exercise self-efficacy with health status and symptoms of depression and anxiety. Furthermore, for health status with functional capacity, dyspnea, pain and depressive symptoms (Table 3). In patients with CHF, significant weak to moderate correlation coefficients were found for health status with symptoms of pain, depression and anxiety, but no other associations were found to be statistically significant within the model (Table 3). In the analysis of the whole group (*n* = 150), significant but mainly weak correlation coefficients were found between most of the variables within the model, but no significant association was found for functional capacity with fatigue and anxiety (Table 3).

**Table 3.** Correlation matrix for variables within the model: for patients with COPD, patients with CHF and the whole patient population.

	Functional capacity	Health status	Dyspnea	Fatigue	Pain	Depressive symptoms	Anxiety symptoms
Patients with COPD, *n* = 60							
Exercise self-efficacy	0.02	0.46*	−0.30	−0.16	−0.36	−0.43*	−0.37*
Functional capacity		0.36*	−0.13	−0.11	−0.19	−0.13	−0.16
Health status			−0.35*	−0.34	−0.49*	−0.45*	−0.35
Patients with CHF, *n* = 60							
Exercise self-efficacy	0.13	0.17	−0.33	−0.35	−0.27	−0.28	−0.19
Functional capacity		0.09	−0.09	−0.15	−0.29	−0.32	−0.27
Health status			−0.15	−0.38	−0.42*	−0.47*	−0.36*
Whole group, *n* = 150							
Exercise self-efficacy	0.26*	0.31*	−0.38*	−0.25*	−0.31*	−0.38*	−0.26*
Functional capacity		0.23*	−0.25*	−0.15	−0.27*	−0.24*	−0.17
Health status			−0.25*	−0.25*	−0.35*	−0.46*	−0.33*

Spearman correlation coefficients between the different variables for patients with chronic obstructive pulmonary disease (COPD), patients with chronic heart failure (CHF) and all patients with COPD and/or CHF.

*Statistically significant correlation coefficients at a *p* < .05 level.

#### Regression analysis

The unadjusted analysis of the group with complete S-ESES scores (*n* = 129) showed that higher age, living alone, a mMRC ≥1, higher level of fatigue, moderate and severe pain, symptoms of depression and anxiety were associated with a lower level of exercise self-efficacy. Patients who were physically active for 30 min or more daily had a better physical capacity, health status and a higher level of exercise self-efficacy. Diagnosis of CHF and/or COPD did not influence the level of exercise self-efficacy, neither did sex or daily smoking. When adjusted for age, sex and diagnosis, the significant associations were unaltered except that age and living alone were no longer found to be significant factors associated with exercise self-efficacy (Table 4).

**Table 4.** Linear regression analysis with exercise self-efficacy score as dependent variable (*n* = 129).

	Regression coefficient, unadjusted (95% CI)	Regression coefficient, adjusted for diagnosis, sex and age (95% CI)
Diagnosis		
Chronic heart failure	Ref	Ref
COPD	2.03 (−1.14–5.20)	0.82 (−2.67–4.32)
COPD and/or CHF	−1.51 (−5.36–2.34)	−2.28 (−6.28–1.73)
Sex		
Men	Ref	Ref
Women	−0.14 (−3.00–2.72)	0.09 (−2.83–3.00)
Age	−0.18 (−0.35– − 0.01)*	−0.18 (−0.37–0.01)
Living alone	−3.15 (−6.04– −0.26)*	−2.75 (−5.82–0.33)
Daily smoking	−0.51 (−4.19–3.17)	−2.86 (−6.91–1.18)
Physical activity		
No	Ref	Ref
15 min/day	1.50 (−2.15–5.15)	1.44 (−2.20–5.07)
30 min/day	4.75 (1.10–8.40)*	5.49 (1.84–9.15)**
>30 min/day mMRC	8.56 (4.55–12.58)***	8.60 (4.59–12.61)***
0	Ref	Ref
1	−4.73 (−8.62– −0.84)*	−4.45 (−8.41– −0.50)*
2	−6.65 (−10.68– −2.63)**	−6.60 (−10.68– −2.52)**
3 + 4	−10.67(−15.45– − 5.59)***	−9.76 (−14.66– −4.87)***
Fatigue total score	−0.71 (−1.25– −0,17)*	−0.87 (−1,41– −0,32)**
Pain		
No pain	Ref	Ref
Slight pain	2.72 (−1.14–6.58)	2.51 (−1.37–6.39)
Moderate pain	−4.07 (−7.72– −0.42)*	−3.87 (−7.52– − 0.21)*
Severe pain	−5.57 (−10.35– −0.79)*	−5.32 (−10.13– − 0.52)*
Depression score (HAD)	−1.00 (−1.43– −0.57)***	−0.98 (−1.42– −0.55)***
Anxiety score (HAD)	−0.62 (−0.95– −0.28)***	−0.65 (−0.10– −0.32)***
6MWD	0.02 (0.007–0.03)**	0.02 (0.003–0.03)*
Health status	0.13 (0.05–0.20)**	0.13 (0.05–0.20)**

Linear regression analysis with exercise self-efficacy score as dependent variable (*n* = 129), unadjusted and adjusted for diagnosis, sex and age. CI: confidence interval; ns: not statistically significant; CHF: chronic heart failure; COPD: chronic obstructive pulmonary disease. mMRC: modified Medical Research Council Dyspnea Scale; HAD: Hospital Anxiety and Depression Scale; 6MWD: six-minute walking distance, health status measured by the Visual Analogue Scale of the EuroQol group.

*p < .05, **p < .005, ***p < .001.

## Discussion

### Statement of principal findings

The primary findings of our study are that patients with COPD and CHF have similar levels of exercise self-efficacy, health status, symptoms and age-adjusted functional capacity. However, the strength of the associations between these variables seems to differ between the two diagnosis groups. Our secondary finding is that dyspnea, fatigue, pain and symptoms of depression or anxiety are important factors that decrease the level of exercise self-efficacy, regardless of diagnosis, sex and age.

### Strengths and limitations

A strength of this study is that this patient sample is representative for patients in primary health care. The age and sex difference and the frequency and level of symptoms in both groups are comparable to those found earlier in Swedish primary care [4]. Even though we have no objective data on severity of COPD and CHF, like forced expiratory volume in one second or left ventricular function, the level of dyspnea and New York Heart Association classification in our study population indicated that few of the patients had an advanced disease stage, and thus they were representative of primary care patient population.

A limitation is that we have no doctor’s diagnosis for those with a double diagnosis. The self-reported data were considered insufficiently reliable, so we did not analyze these patients as a separate group. Another limitation of this study is the relatively small number of patients in the separate diagnosis groups, which probably caused that only few significant and mainly weak correlation coefficients were found within the model for COPD and CHF separately. The fact that more and stronger significant correlation coefficients were found in the group as a whole strengthens this assumption and indicates that our conceptual model is applicable for both COPD and CHF patients. The fact that we found less and weaker associations within the model for patients with CHF might, on the other hand, indicate that other factors, like comorbidity and social and environmental factors, that are not included in our model, play a larger role in patients with CHF [26,27]. However, the regression analysis showed an association between exercise self-efficacy and all variables that were included in our model, regardless of diagnosis. Few other studies have compared patients with COPD and CHF, but similarities and differences between the two disease groups have been found before in a study exploring the relationship between dyspnea, functional status and health status in patients with COPD and CHF [28]. The strength and differences in significant correlation coefficients between the variables for patients with COPD and CHF, that probably are even present at an individual level, stress the need of offering several suitable strategies for the same problem when offering joint self-management group education.

### Findings in relation with other studies and meaning of the study

This is, to our knowledge, the first study comparing levels of exercise self-efficacy between patients with COPD and CHF in primary health care. Self-efficacy in patients with COPD and CHF has been compared before, but in a hospital-selected patient group and using a scale including a broader variation of behavior related to health [28]. Our results are in line with this study’s result: that patients with COPD and CHF have a comparable level of self-efficacy.

A clinically important finding is that dyspnea, fatigue, pain and depressive and anxiety symptoms influence the level of exercise self-efficacy. Focus is often on dyspnea and fatigue, as these symptoms are directly linked to the pathophysiology of both COPD and CHF. However, pain, depression and anxiety are not directly linked to the pathophysiology of the diseases but present in many patients with COPD and CHF and are often undertreated [29]. We found that moderate to severe pain is associated with reduced exercise self-efficacy. Pain has been shown to cause physical inactivity, functional limitations and lower health status in both patients with COPD and CHF [26,30]. Also, anxiety and depression have been found to have a negative influence on a patient’s self-efficacy [31,32]. On the other hand, it has been shown that increased physical exercise will have a positive effect on levels of pain and depressive symptoms [33]. The association between exercise self-efficacy and symptoms might be bi-directional or be mediated by other variables, like physical exercise itself. Most patients in our study reported symptoms of depression and anxiety under the level that could indicate a diagnosis of depression or anxiety disorder, but it seems that even symptoms at this low level are negatively associated with the patient’s self-efficacy. A recent study showed that issues like psychological well-being, stress and fatigue often are underemphasized in self-management education [34]. Discussing these issues and supporting the patients in finding strategies will possibly facilitate rehabilitation efforts.

In our study, age was found to be associated with the level of exercise self-efficacy in the unadjusted regression analysis but not after adjustment for sex and diagnosis. We found though that functional capacity decreased with age, which is in line with prior studies [35], and that the level of exercise self-efficacy was associated with functional capacity. So even though we did not find that the level of exercise self-efficacy was associated with age, it is easy to imagine that ‘modeling’ is more effective in a group of patients at the same stage of life and with the same functional capacity.

This study focused on the similarities between patients with COPD and CHF. There are, of course, differences between these patient groups, including, for example, the use of diuretics or sensitivity to cold or damp air, that need different coping strategies and make ‘modeling’ more difficult. Further research on effectiveness of joint self-management groups and qualitative research on patient and care-giver experiences is needed for further evaluation of the feasibility of these groups.

In summary, the similar levels of exercise self-efficacy, symptoms, functional capacity and health status that we found in patients with COPD and CHF create the conditions for a beneficial effect of ‘modeling’ when utilized in a self-management group with a focus on increasing exercise self-efficacy. When forming self-management groups with a focus on exercise self-efficacy, it seems more relevant to consider the level of symptoms than the specific diagnosis of COPD or CHF.
